# Prolonged Time From Symptoms to Diagnosis Is Associated With an Inferior Progression‐Free Survival in Diffuse Large B‐Cell Lymphoma

**DOI:** 10.1002/cam4.71409

**Published:** 2025-11-23

**Authors:** Susanna Tokola, Katja Marin, Milla E. L. Kuusisto, Hanne Kuitunen, Marjukka Pollari, Sirkku Jyrkkiö, Minna Suominen, Kristiina Vuolukka, Minna Harmanen, Kaisa Sunela, Aino Rönkä, Tuomas Selander, Annikki Aromaa‐Häyhä, Stella Ylhäinen, Tuula Klaavuniemi, Anna Hakalahti, Outi Kuittinen

**Affiliations:** ^1^ Department of Radiotherapy and Oncology Oulu University Hospital Oulu Finland; ^2^ Cancer Center Kuopio University Hospital Kuopio Finland; ^3^ University of Eastern Finland Kuopio Finland; ^4^ Department of Internal Medicine Länsi‐Pohja Central Hospital, Kemi, the Wellbeing County of Lappland Kemi Finland; ^5^ Biomedicine and Internal Medicine Research Unit University of Oulu Oulu Finland; ^6^ Department of Oncology Tampere University Hospital, TAYS Cancer Center Tampere Finland; ^7^ The Wellbeing Services County of Southwest Finland Turku University Hospital Turku Finland; ^8^ North Karelia Central Hospital, Cancer Center Joensuu Finland; ^9^ Finnish Medicines Agency Tampere Finland; ^10^ Department of Oncology Kainuu Central Hospital Kajaani Finland

**Keywords:** diagnostic pathways, diffuse large B‐cell lymphoma, international prognostic index (IPI), progression‐free survival, proliferating tumors

## Abstract

**Introduction:**

Diffuse large B‐cell lymphoma (DLBCL) is a heterogeneous disease, with often a high Ki‐67 proliferation index. Prognosis is associated with lymphoma stage, lactate dehydrogenase level, and metabolic tumor volume. Thus, intuitively, time from symptom onset to diagnosis would be assumed to be essential for treatment outcome, but existing literature is conflicting.

**Materials and Methods:**

This prospective study evaluated diagnostic pathways and their impact on treatment outcomes in 160 patients with DLBCL.

**Results:**

The mean time from symptom onset to treatment initiation (TST) was 146 days. Mean patient‐associated delay from the onset of symptoms to the first healthcare contact was 54 days; mean time from symptoms to biopsy was 130 days; and from biopsy to treatment initiation was 19 days. Prolonged time from symptom onset to treatment (TST) > 3 months was associated with a higher International Prognostic Index (IPI) score, whereas prolonged time from biopsy to treatment initiation (TBT) > 2 weeks was associated with better performance status and a lower IPI score. Prolonged time from symptom onset to treatment initiation was not associated with progression‐free survival (PFS). Prolonged time from symptom onset to diagnostic biopsy > 7 weeks implied inferior progression free survival in the whole study cohort (2 year PFS 89% vs. 74%, *p* = 0.012), as well as among patients with highly proliferating tumors with Ki67 > 70% (2 year PFS 93% vs. 63%, *p* < 0.001). Longer time from biopsy to treatment initiation (TBT) > 2 weeks implied better progression‐free survival (PFS) in patients with low proliferating tumors (2 year progression‐free survival (PFS) 25% vs. 87%, *p* = 0.032), respectively.

## 1 Introduction

Diffuse large B‐cell lymphoma (DLBCL) is the most common lymphoma subtype [1, 2]. Its incidence is increasing in Western countries [3], mainly because of aging. Despite therapeutic improvements, a considerable number of patients do not achieve long‐lasting responses and need more intensive and expensive salvage therapy options, and a proportion of them also succumb to their disease [4, 5].

Despite being biologically heterogeneous, many DLBCL cases have a high tumor proliferation rate, and patients suffer from rapidly growing tumors and a declining performance status. For decades, the International Prognostic Index (IPI) has been used to delineate patients into different prognostic subgroups [6]. The four individual factors (tumor stage, number of extranodal disease sites, lactate dehydrogenase level (LDH), and performance status) in this five‐point scoring index worsen during disease progression. Moreover, in recent years several studies have shown that total metabolic tumor volume measured by baseline positron emission tomography/computed tomography (PET/CT) scan is associated with a higher risk of progression and disease‐associated death [7, 8, 9, 10]. For these reasons, it could be anticipated that earlier diagnosis would lead to better response rates and improved disease‐free and overall survival (OS). Considering the increasing costs from salvage therapies such as chimeric antigen receptor T‐cell (CAR‐T) cell therapy, we assume that earlier diagnosis would also reduce the disease‐associated socioeconomic burden.

The existing literature concerning diagnostic delays in DLBCL is sparse and controversial. Nikonova et al. did not find any association between total diagnostic delay and treatment outcome [11], whereas in two studies, excessive diagnostic delay (time from first symptoms to diagnosis > 6 months) predicted worse overall survival [12], or progression‐free survival [13].

DLBCL is a heterogeneous disease with a wide variation in tumor proliferation rates. Although Ki‐67 has an unclear prognostic significance [14, 15, 16], it can be assumed that patients with the highest tumor proliferation also have the most promptly progressing symptoms and may therefore seek medical attention earlier than those with more indolent disease. This has been shown in earlier studies, which found a short diagnosis‐to‐treatment interval (DTI) to be associated with poor prognostic features and adverse outcomes [17, 18, 19, 20, 21].

The primary aim of the present study was to evaluate, in a prospective setting, the diagnostic delays of patients with DLBCL diagnosed in seven Finnish hospitals by dividing the course of delays into patient‐associated delays, delays associated with primary health care, and delays associated with secondary health care [22]. The secondary aim was to evaluate the impact of these delays on treatment outcomes in the entire patient cohort and in subgroups stratified according to tumor proliferation rate.

## 2 Materials and Methods

This multicenter study included patients aged 18 years or older at diagnosis and a newly diagnosed DLBCL. The patients were enrolled between October 2016 and March 2020 in seven hospitals in Finland: four university hospitals and three central hospitals. The recruited patients completed a questionnaire describing their socioeconomical background, primary disease symptoms, and time periods from the onset of symptoms to contacts with different levels of the healthcare system during their diagnostic and treatment processes. The entire content of the questionnaire is presented in the Supporting Information. Clinical data, including comprehensive diagnostic pathways in secondary healthcare as well as clinical disease presentation, lymphoma treatments, and disease outcome, were collected from patient records and analyzed together with the data from the questionnaires.

Timelines during diagnostic pathways were calculated based on dates provided in a questionnaire. The time from symptom onset to treatment (TST) was calculated from the first day that the patient reported symptoms to have started, to the first day of lymphoma treatment, including also prephase with corticosteroids w/o chemotherapy. The time from symptom onset to biopsy (TSB) was calculated from the first day that the patient reported symptoms to have started to the date of diagnostic biopsy. The time from biopsy to treatment (TBT) was calculated from the date of diagnostic biopsy to the first date of lymphoma treatment. Timelines were compared with age at lymphoma diagnosis, sex, lymphoma stage, B symptoms, IPI score, World Health Organization (WHO) status, and proliferation rate (Ki‐67 proliferation index) to find out correlations between diagnostic timelines and prognostic factors.

Cut‐off values of the timelines were analyzed with a ROC curve. For analyses, the following cut‐off values were used: time from symptom onset to treatment initiation (TST) 3 months, time from symptom onset to biopsy (TSB) 7 weeks, and time from biopsy to treatment initiation (TBT) 2 weeks. Proliferation rate was classified as high at a cut‐off value of over 70%. PFS was calculated from the date of the pathological diagnosis to the date of disease progression, death, or the last date of follow‐up, whichever came first.

This study was approved by the ethics committee of the North‐Ostrobothnia Health Care District and was conducted according to the Good Clinical Practice guidelines and the Declaration of Helsinki. All patients included in the study provided informed consent before completing the questionnaire.

Statistical analyses were performed using IBM SPSS Statistics software (version 29.0.1.0). The chi‐squared test was used to evaluate the correlation between variables, and the Kaplan–Meier and log‐rank tests were used for survival analyses.

## 3 Results

Patient demographics according to time from symptom onset to treatment initiation (TST) groups are presented in Table 1. Informed consent and data from the questionnaires were received from 170 patients, but 10 patients were excluded from the analysis because of central nervous system lymphoma localization only or a final diagnosis other than DLBCL (T‐cell lymphomas, lymphoblastic lymphoma, acute myeloid leukemia, and marginal zone lymphoma), leaving 160 patients for final analysis. The mean age at diagnosis was 65 (range 28–95) years; 56% of patients were male and 44% were female.

**TABLE 1:** Patient demographics according to TST: Three months or less versus more than three months.

	TST 3 months or less	TST over 3 months	*p*
Age mean (range), years	63 (34–87)	68 (28–95)	0,019
Sex			ns
Male	52 (55%)	38 (58%)	
Female	42 (45%)	28 (42%)	
Stage *			ns
1	17 (18%)	13 (20%)	
2	20 (21%)	12 (19%)	
3	22 (23%)	11 (17%)	
4	35 (37%)	28 (44%)	
B‐symptoms			ns
Yes	20 (22%)	17 (26%)	
No	72 (78%)	49 (74%)	
WHO performance status			ns
0	29 (31%)	20 (32%)	
1	35 (38%)	22 (35%)	
2	20 (22%)	14 (22%)	
3	7 (8%)	6 (10%)	
4	2 (2%)	1 (2%)	
IPI score			ns
0	6 (6%)	1 (2%)	
1	18 (19%)	17 (27%)	
2	23 (24%)	17 (27%)	
3	25 (27%)	15 (23%)	
4	14 (15%)	12 (19%)	
5	8 (9%)	2 (3%)	
Proliferation rate			
High, > 70%	70 (89%)	37 (74%)	0,032
Low, < 70%	9 (11%)	13 (26%)	

* According to Ann Arbor classification.

Two patients were lost from follow‐up, and in three additional patients, their follow‐up status could not be confirmed at the time of data collection. Median follow‐up time was 35 (range 0–72) months. At the end of the follow‐up, 120 patients were alive in ongoing remission, and 33 patients had died.

### 3.1 Diagnostic Pathways

The healthcare units of the first healthcare contacts are described in Table 2. Over half of the patients contacted their primary health care provider at the health services center first, either the emergency care (24%) or a doctor's nonurgent appointment (30%). Five percent of the patients first contacted a secondary health care emergency unit. In all, 12% of the patients had their first medical consultation on the same day as the symptom onset, and 53% of patients had their first medical consultation within 2 weeks of the symptom onset.

**TABLE 2:** Healthcare unit of the first healthcare contact.

Unit of the first healthcare contact	*n* = 156	%
Health services center, emergency care	39	24
Secondary health care, emergency unit	8	5
Health services center, doctor's appointment	48	30
Occupational health care	19	12
Private health service	20	13
Some other	22	14

In all, 20% of the patients were evaluated as “needing immediate investigations”, and 33% “needing investigations within the next 1‐7 days”. However, 10% of the patients were classified as “no need for further investigations” based on their first contact. Forty percent of patients reported that their symptoms had been treated as other diseases than lymphoma.

### 3.2 Timelines of Diagnostic

The mean durations of patient‐reported diagnostic steps are presented in Table 3. Mean time from symptom onset to the initiation of treatment was 146 (range 7–1124) days.

**TABLE 3:** Timelines during the diagnostic pathway.

Timeline	Mean (days)	Range
Time from the onset of symptoms to the first contact with healthcare	54	0–1065
Time from the first contact to the first appointment with a doctor	9	0–372
Time from the first doctor's appointment to referral	36	0–504
Time from referral to the first contact in the hospital	11	0–183
Time from the first contact in the hospital to histological diagnosis by a pathologist	19	−34–158
Time from histological diagnosis by a pathologist to the patient information	2	−10–60
Time from patient information to the first appointment with a hematologist/oncologist	6	−29–100
Time from the first appointment with the hematologist/oncologist to the treatment initiation	5	0–64
Time from the onset of symptoms to the treatment initiation (TST)	146	7–1124
Time from onset of symptoms to diagnostic biopsy (TSB)	130	−1108
Time from the biopsy to the treatment initiation (TBT)	19	0–137

It took a mean of 54 days from the onset of symptoms to the first contact with the healthcare system. From this first contact with primary care, it took a mean of 45 days to the first visit to a secondary care hospital, and an additional 22 days to the date when the patient reported hearing the diagnosis of the lymphoma for the first time. From this date, it took 6 days to the first visit in the oncology department and, thereafter, 5 days to the initiation of treatment. For 54% of the patients, TST was more than 3 months.

### 3.3 Association of Diagnostic Timelines With Baseline Characteristics

Patients with TST below 3 months were younger, 63 versus 68 years (*p* = 0.006), and more often had a high Ki‐67 proliferation index, 89% versus 74% (*p* = 0.007), than patients with TST over 3 months.

Patients with TBT below 2 weeks had a higher Ki‐67 proliferation index level (*p* = 0.007) and unfavorable World Health Organization (WHO) and IPI profiles. In contrast, patients with TSB below 7 weeks had a more favorable IPI scores (Table 4). TSB below 7 weeks was associated with a shorter TST (*p* < 0.001) and shorter patient‐associated delay (*p* < 0.001) but not with shorter TBT.

**TABLE 4:** WHO performance status and IPI score comparison in patients with time from biopsy to treatment (TBT) 2 weeks or less versus over 2 weeks, and time from symptom onset to biopsy (TSB) 7 weeks or less versus over 7 weeks.

	TBT 2 weeks or less	TBT > 2 weeks	*p*	TSB 7 weeks or less	TSB > 7 weeks	*p*
WHO performance status	*n* (%)	*n* (%)	< 0.001	*n* (%)	*n* (%)	0.888
0	9 (14%)	39 (44%)		28 (33%)	21 (30%)	
1	20 (32%)	35 (40%)		32 (38%)	25 (35%)	
2	24 (38%)	9 (10%)		18 (21%)	16 (22%)	
3	7 (11%)	6 (7%)		6 (7, 1%)	7 (10%)	
4	3 (5%)	0 (0%)		1 (1%)	2 (3%)	
IPI score			0.011			0.037
0	2 (3%)	5 (6%)		5 (6%)	2 (3%)	
1	8 (13%)	27 (30%)		23 (27%)	12 (17%)	
2	11 (18%)	26 (29%)		17 (20%)	23 (32%)	
3	21 (33%)	19 (21%)		27 (31%)	13 (18%)	
4	15 (24%)	11 (12%)		9 (10%)	17 (24%)	
5	6 (10%)	3 (3%)		5 (6%)	5 (7%)	

### 3.4 Impact of the Diagnostic Timelines on Treatment Outcome

#### 3.4.1 Total Delay

**FIGURE 1:**
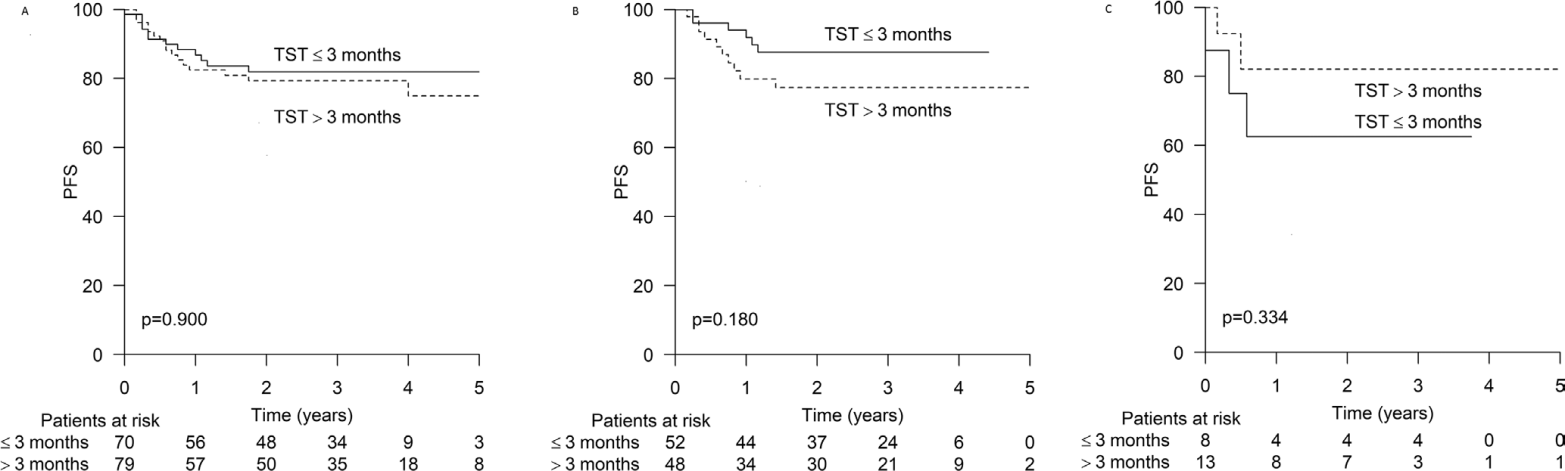
Progression‐free survival (PFS) according to time from symptoms to diagnosis. (A) All patients (*n*: 149). (B) Patients with high proliferation index (*n*: 100). (C) Patients with low proliferation index (*n*: 21).

In the entire study population, there was no difference in progression‐free survival (PFS) between patients who started treatment during the first 3 months after the onset of symptoms or later (Figure 1A). When analyzing treatment outcome stratified by proliferation rate (low; Ki‐67 proliferation index less than 70% vs. high Ki‐67 proliferation index 70% or higher), we found a trend toward improved PFS among patients with a high Ki‐67 proliferation index and TST below 3 months. The 2‐year PFS rate was 88% versus 77%, respectively (*p* = 0.180) (Figure 1B). In contrast, among those with a low Ki‐67 proliferation index and short TST, the 2 year PFS was 62% versus 82% in patients with TST > 3 months (*p* = 0.334) (Figure 1C).

#### 3.4.2 Time From Biopsy to Treatment Initiation (TBT)

**FIGURE 2:**
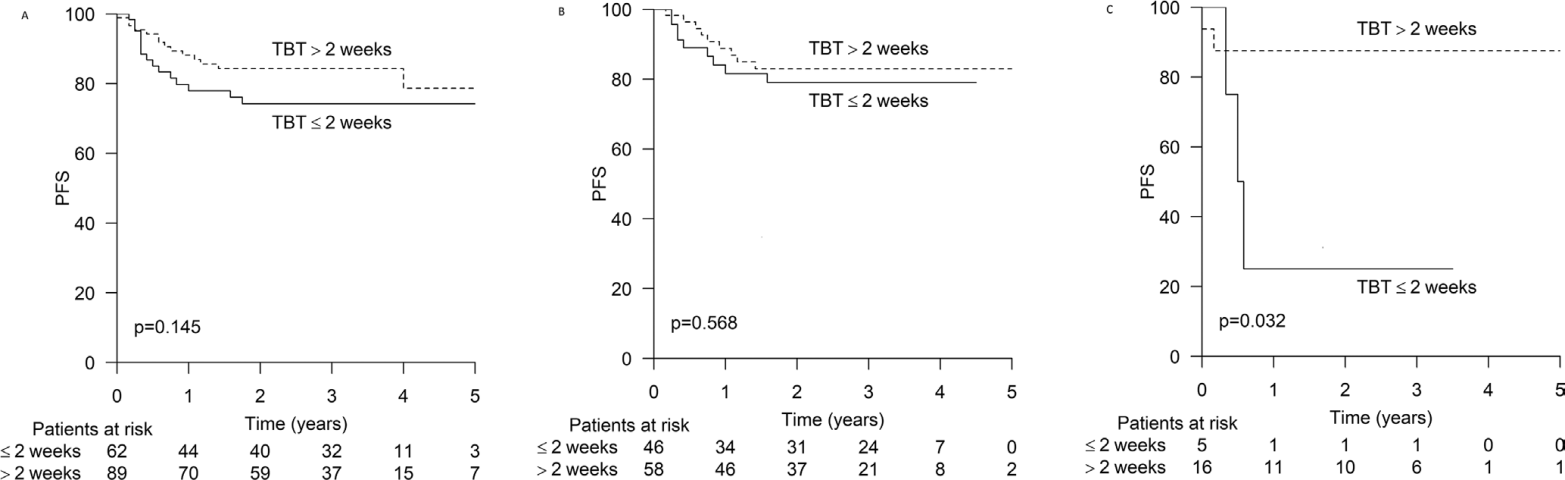
Progression‐free survival (PFS) according to time from biopsy to therapy. (A) All patients (*n*: 151). (B) patient with high proliferation index (*n*: 104). (C) Patients with low proliferation index (*n*: 21).

In the entire population, there was a trend toward an adverse outcome among patients with TBT less than 2 weeks (*p* = 0.145) (Figure 2A). Among patients with a high Ki‐67 proliferation index, no difference in progression‐free survival (PFS) was detected according to TBT (*p* = 0.568) (Figure 2B). Patients with a low Ki‐67 proliferation index and TBT less than 2 weeks had an inferior PFS at 2 years: 25% versus 87%, respectively (*p* = 0.032). HR for progression or death was 7.74 (CI 0.948–34.869) (Figure 2C).

#### 3.4.3 Time From Symptoms to Biopsy (TSB)

**FIGURE 3:**
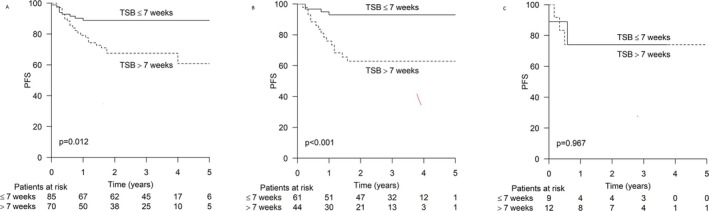
Progression‐free survival (PFS) according to time from symptoms to biopsy. (A) All patients (*n*: 155). (B) Patients with high proliferation index (*n*: 105). (C) Patients with low proliferation index (*n*: 21).

Shorter TSB, 7 weeks or less, was associated with a favorable outcome in an unselected population. Two‐year PFS was 89% for the shorter TSB group versus 74% for the longer TSB group (*p* = 0.012) (Figure 3A).

When considering the proliferation rate, the difference in PFS was even more pronounced among patients with a high proliferation index. The 2 year PFS was 93% versus 63% (*p* < 0.001) (HR: 5.892 CI: 1.953–17.772) in favor of shorter TSB (Figure 2B). No difference in PFS was found among patients with a low Ki‐67 proliferation index (Figure 3C).

## 4 Discussion

Here, we report a prospective study demonstrating the association between different diagnostic timelines and disease outcome in DLBCL. This is the first study to describe the complete diagnostic pathway of patients, starting from symptom onset to the initiation of lymphoma treatment and up to long‐term follow‐up. In addition, this is the first study to also consider tumor aggressiveness measured by proliferation rate with respect to the above‐mentioned parameters. We found that a longer time from the onset of symptoms to diagnostic biopsy was associated with adverse PFS in the unselected population, as well as among patients with high Ki‐67 proliferation index tumors, but not in those with low Ki‐67 proliferation index tumors. In line with previous data [19, 20], a short time from biopsy to treatment initiation demonstrated a trend toward decreased PFS. This association was most prominent among patients with slowly proliferating tumors.

The treatment results for DLBCL have improved over the last two decades [23]. The cyclophosphamide, doxorubicin, vincristine, and prednisolone (CHOP) treatment was adopted as the standard of care in the 1980s. Incorporation of the cluster of differentiation 20 (CD20) antibody rituximab into therapy at the end of the 1990s further improved outcomes. By contrast, until recently, the prognosis of patients with relapsed and refractory disease has been dismal, and only a few transplant‐eligible patients with a positive response to salvage therapy have survived [24]. Currently, there is a dawn of new treatment options, and an increasing number of patients with relapsed or refractory disease can be salvaged with CAR‐T‐cell therapy or novel antibody therapies [25, 26]. The drawbacks of these new therapies are their extremely high costs and the risk of prolonged side effects, such as cytopenia and hypogammaglobulinemia [27]. Additionally, relapsing disease increases patients' emotional stress and suffering. All this highlights the importance of optimizing primary therapy outcomes. Here, we describe that early diagnosis is an integral part of successful treatment. This effect was most prominent in patients with highly proliferating tumors.

DLBCL is an aggressive tumor with often a high Ki‐67 proliferation index. If left untreated, most patients die of their disease in a few months. The risk of lymphoma‐related death is associated with IPI risk factors, including age, tumor stage, lactate dehydrogenase level, number of extranodal lesions, and patient performance status [6]. Furthermore, high total metabolic tumor volume at diagnosis increases the risk of relapse and disease‐associated death [7, 8, 9, 10]. These factors worsen over the course of weeks or months before the diagnosis. Concerning the high Ki‐67 proliferation index of DLBCL, it may be assumed that an early diagnosis would be essential for survival.

Four previous studies have analyzed the association between diagnostic delays and treatment outcomes [17, 18, 19, 20]. These studies (Maurer et al. with 2430 patients, Olszewski et al. with 104,465 patients, Camus et al. with 345 patients, and Yoshida et al. with 199 patients) reported paradoxically that patients with a short diagnosis‐to‐treatment interval (DTI) were associated with worse treatment outcomes. This is probably explained by the fact that short DTI was also associated with an advanced‐stage disease, and an adverse prognostic profile. We confirmed this finding. In our study, patients with TBT below 2 weeks had an adverse risk profile and worse PFS. We find this logical as patients with severe symptoms and decreased performance status are expedited through a diagnostic work‐up and treatment initiation, resulting in a shorter time from diagnosis to treatment, which is still unable to turn over the effect of adverse prognostic factors.

However, time from diagnosis to treatment initiation constitutes only a small proportion of the total delay. In our study, this time covered 19 days out of a total of 146 day timeline, implying that to evaluate the impact of delays, we should evaluate the entire diagnostic process, starting from symptom onset to treatment initiation. Three previous studies by Nikonova et al. [11] (with 278 patients), Zurko et al. [12] (with 104 patients), and Xavier et al. [13] (with 42 patients) have evaluated the impact of time from symptom onset to treatment initiation (TST) on disease outcome. Both Zurko and Xavier found an association with inferior outcome and a diagnostic delay of over 6 months; however, this was not demonstrated in the study by Nikonova et al. In our study, TST longer than 3 months was not associated with PFS in the entire cohort. Among patients with a high Ki‐67 proliferation index tumor, there was a trend toward an adverse outcome with a longer TST.

When analyzing the time from symptom onset to diagnostic biopsy, a delay of 7 weeks or longer was associated with poor PFS both in the unselected population as well as in the high Ki‐67 proliferation index population, but not in the low Ki‐67 proliferation index population. Patients with longer TSB presented with higher IPI scores. Time from symptom onset to biopsy had an inverse correlation with the time from biopsy to treatment initiation.

Our results can be explained logically as follows. The time from symptom onset to biopsy reflects the time before diagnosis of an aggressive lymphoma is set. During this period, there may be cases of misdiagnosis of a benign disease or erroneous estimation of the seriousness of the situation. This may lead to a prolonged diagnostic pathway during which the patients' disease progresses and the IPI score worsens. However, after being diagnosed, those in the worst situation proceed most promptly to treatment initiation, thus partly counteracting the effects of prolonged time from symptom onset to biopsy and thus diluting the impact of time from symptom onset to treatment initiation.

Mean patient‐related delay, which refers to the time from symptom onset to the first contact with healthcare, was 54 days out of a total delay of 146 days. This suggests that most delays are related to the performance of primary and secondary healthcare systems. The issue in primary healthcare may be that clinicians do not recognize early enough that patient symptoms might be a sign of lymphoma or that the waiting times for diagnostic work‐up are too long. Lymphoma may arise from any human organ and has a wide variety of clinical presentations, challenging early diagnosis. An in‐depth analysis of the reasons for delays in primary healthcare is warranted to improve early diagnostics. In secondary healthcare, the suspicion of lymphoma already exists. In our study, it took a mean of 11 days from writing a referral to the first visit to a hospital, followed by 19 days to obtain a pathology report, 2 days to notify the patient about the biopsy result, 6 days to the first appointment with an oncologist, and thereafter 5 days until treatment initiation. These delays could be avoided by informing all medical professionals of the urgent nature of treatment initiation in aggressive lymphomas.

The advantages of our study include its prospective setting and the fact that patient‐reported questionnaires enabled us to uncover patient‐related and primary care‐related delays. We also analyzed separately each time point during the patient's diagnostic pathway. Another important issue is that we collected data regarding the tumor proliferation rate, which is a potential source of bias in studies of heterogeneous diseases such as DLBCL. This may be one of the reasons that made it possible for us to prove the adverse prognostic impact of delayed diagnosis, which was most prominent among cases with a high Ki‐67 proliferation index. We had several clinicians in seven centers recruiting the study patients; thus, these patients do not represent all consecutive patients treated during the study period. Although unlikely, this raises the possibility of selection bias in patient recruitment, interfering with the results.

The biggest limitation of the study is our sample size, which did not allow us to perform in‐depth analyses of tumors with variable proliferation rates. Our sample size also forced us to use a single cut‐off value in the survival analyses. This does not necessarily mean that these time points in the patient pathway should be used to describe optimal diagnostic timelines; instead, it seems evident that there probably is a linear association between delays and outcomes. These optimal timelines are likely dependent on DLBCLs' growth rate. However, the proliferation rate of the tumor is not yet known during the diagnostic pathway. Therefore, more research is needed to create clear‐cut recommendations for the optimal timing of diagnostic steps.

In conclusion, we found that, especially among patients with high proliferation rate DLBCL, delayed time from symptom onset to biopsy is associated with an adverse disease risk profile and decreased PFS. After biopsy, those patients with the worst disease presentation have the shortest time to treatment initiation. A factor still insufficient to fully counteract the effect of the adverse risk profile. To save societal costs and individual suffering, it would be integral to speed up the early disease diagnostics and treatment initiation in the case of lymphoma suspicion.

## Author Contributions

**Susanna Tokola:** conceptualization, investigation, writing – original draft, methodology, validation, writing – review and editing, formal analysis, data curation. **Katja Marin:** investigation, data curation, writing – original draft, formal analysis. **Milla E. L. Kuusisto:** conceptualization, investigation, writing – original draft, formal analysis. **Hanne Kuitunen:** investigation, writing – original draft, formal analysis, writing – review and editing. **Marjukka Pollari:** investigation, writing – review and editing. **Sirkku Jyrkkiö:** investigation, writing – review and editing. **Minna Suominen:** investigation, writing – review and editing. **Kristiina Vuolukka:** investigation, writing – review and editing. **Minna Harmanen:** investigation, formal analysis, writing – review and editing. **Kaisa Sunela:** methodology, conceptualization, investigation, writing – review and editing, formal analysis. **Aino Rönkä:** writing – review and editing, investigation, formal analysis. **Annikki Aromaa‐Häyhä:** investigation, writing – review and editing, formal analysis. **Stella Ylhäinen:** formal analysis, writing – review and editing. **Tuula Klaavuniemi:** investigation, conceptualization, writing – review and editing, formal analysis. **Anna Hakalahti:** investigation, writing – review and editing. **Tuomas Selander:** formal analysis, visualization. **Outi Kuittinen:** conceptualization, investigation, writing – original draft, formal analysis, methodology, writing – review and editing, project administration, resources, data curation.

## Funding

There are no funding sources to declare.

## Conflicts of Interest

Outi Kuittinen, Research grant: Incyte. Consultation fees: Sobi, Janssen, Abbvie. Advisory board membership: Abbvie, Serb SA, Roche.

## Supporting information


**Data S1:** Supporting Information.

## Data Availability

The data that support the findings of this study are available on request from the corresponding author. The data are not publicly available due to privacy or ethical restrictions.
