# Supplementary material for: Prolonged Time From Symptoms to Diagnosis Is Associated With an Inferior Progression‐Free Survival in Diffuse Large B‐Cell Lymphoma
Source: Cancer Med. 2025 Nov 23;14(22):e71409. doi: 10.1002/cam4.71409 (PMC12640614; doi:10.1002/cam4.71409)
Supplement: Supplementary file 1 — Data S1: Supporting Information. [file CAM4-14-e71409-s001.docx]

PERSONAL DATA

Social security number _______________

| Gender |
| --- |
| Female |
| Male |
| Other |
| I don’t want to answer   \| Is your marital status... (Please choose only one of these options.) \| \| --- \| \| Single \| \| Married \| \| Cohabitation \| \| Registered partnership \| \| Judicial separation \| \| Divorced \| \| Widow \| \| Other  How many persons live in your household? Altogether _____ persons \| |

| Basic education |
| --- |
| Primary school (in Finland until 1970’s) |
| Comprehensive school (or first five forms of secondary; in Finland until 1970’s) |
| General upper secondary school |
| No basic education |

| Occupational education |
| --- |
| No occupational education |
| Short occupational education, occupational course, or training |
| Vocational school |
| Occupational education after upper secondary school (University of applied sciences etc) |
| University |

| Professional group, which is most applicable to You (Choose only one, please) |
| --- |
| Director, executive, leading position |
| Senior officer or manager |
| Functionary |
| Employee / worker |
| Entrepreneur or freelancer |
| Farmer |
| Student |
| Retiree |
| Housewife or househusband |
| Unemployed  Other                             \| Your monthly gross income (If not sure precisely, an estimate) \| \| --- \| \| under 500 EUR \| \| 500-1499 EUR \| \| 1500-2499 EUR \| \| 2500-2999 EUR \| \| 3000-4999 EUR \| \| 5000-6499 EUR \| \| over 6500 EUR \| |
| \| Your health care district is \| \| --- \| \| South Karelia \| \| North Karelia \| \| South Ostrobothnia \| \| Central Ostrobothnia \| \| North Ostrobothnia \| \| South Savo \| \| North Savo \| \| East Savo \| \| Kainuu \| \| Kanta-Häme \| \| Central Finland \| \| Kymenlaakso \| \| Länsi-Pohja \| \| Pirkanmaa \| \| Lapland \| \| Satakunta \| \| Ostrobothnia \| \| Southwest Finland \| \| Helsinki and Uusimaa \| |

| Population of your municipality of residence (If you are not sure precisely, an estimate) |
| --- |
| under 2000 |
| 2000-4999 |
| 5000-9999 |
| 10-19 999 |
| 20 000 – 49 999 |
| 50 000 – 99 999 |
| over 100 000 |

SYMPTOMS AND TIME OF ONSET OF SYMPTOMS

| 1. Onset of symptoms |
| --- |
| 1.1. What was the time for symptom onset?  ___________Date (If not sure precisely, an estimate, for example the month and the year, when the symptoms first began) |
| 1.2. What were the first symptoms? |
| chills/fevering |
| night sweats |
| weight loss |
| fatigue or abnormal exhaustion |
| indolent lump or lumps  If you noticed a lump or lumps, where were they?  _____________________________________________________ |
| Other symptoms, specify? _____________________________________  ______________________________________________________  ______________________________________________________ |

| 1. Time from symptom onset to first contact to health care system?   _________________________ Date of first contact (If you remember the date) |
| --- |
| Immediately on the same day |
| 1 - 2 days |
| 3 - 7 days |
| 1- 2 weeks |
| 3 - 4 weeks |
| 1 - under 2 months |
| 2 – 6 months  over 6 months  I had no symptoms |

| 1. The first contact to health care system |
| --- |
| - 1. What was the first health care unit You first contacted? |
| Health services centre, emergency care |
| Secondary health care, emergency unit |
| Health services centre, doctor’s appointment |
| Occupational health care |
| Private health services |
| Some other, what? ____________________________________ |
|  |
| - 1. How was the first evaluation of your symptoms made? |
| Counseling by phone (no appointment) |
| Referral to nurse appointment |
| Referral to doctor’s appointment |
| Some other, how? _________________________________________ |

| 1. Which were the symptoms that made you to contact health care? |
| --- |
| You can write below informally.  _______________________________________________________ |
| _______________________________________________________ |

FIRST APPOINTMENT IN HEALTH CARE

| 5. When was the first appointment in health care after the first contact? |
| --- |
| _____________ First nurse appointment, date |
| _____________ First doctor’s appointment, date |
|  |
| If You don’t remember precisely, an estimate |
| Nurse appointment |
| My first contact was a nurse appointment |
| Immediately on the same day |
| 1 – 2 days after the first contact |
| 3 – 7 days after the first contact |
| 1 – 2 weeks after the first contact |
| 3 – 4 weeks after the first contact |
| 1 – 2 months after the first contact |
| In 6 months after the first contact |
| over 6 months after the first contact |
| Other, when? ________________ |
|  |
| Doctor’s appointment |
| My first contact was a doctor’s appointment |
| Immediately on the same day |
| 1 – 2 days after the first contact |
| 3 – 7 days after the first contact |
| 1 – 2 weeks after the first contact |
| 3 – 4 weeks after the first contact |
| 1 – 2 months after the first contact |
| In 6 months after the first contact |
| over 6 months after the first contact |
| Other, when? _________________ |

| 1. What was the conclusion of the first doctor’s appointment? |
| --- |
| No need for further investigations |
| Non-urgent investigatios indicated |
| Urgent investigations indicated (for 1-7 days) |
| Emergency investigations indicated (on the same day or at least the next day) |
| Some other, what? ___________________ |

| \| 7. Did you make an agreement for a control at the first doctor’s appointment? \| \| --- \| \| No \| \| Yes \| \| If yes, on which way? \| \| contact by phone \| \| a new appointment to a doctor \| \| if symptoms continue, you should take contact to health care again \| \| Some other way, how? _______________ \|  \| 8. Was there any further investigations based on the first doctor appointment? \| \| --- \| \| No \| \| Yes \| |
| --- | --- | --- | --- | --- | --- | --- | --- | --- | --- | --- | --- |
| If yes, what? Please, also fill the date. In case you don’t remember the exact date, please make an estimate. |
| laboratory tests _______________ date |
| X-ray examinations _______________ date |
| ultrasound examinations _______________ date |
| biopsy _______________ date |
| \| 9. Were your symptoms treated as some other disease? \| \| --- \| \| Yes \| \| No \| \| If yes, what kind of treatment did you got and for which disease?  ___________________________________________________________________________________________________________________________________ \|  \| \| 10. How many contacts to health care did you have before referral to specialized medical care? \| \| --- \| \| Phone calls ____ pcs \| \| Nurse appointments ____ pcs \| \| Doctor appointments ____ pcs \|   11. Referral to specialized medical care, date ____________________________ \| \| --- \| --- \| --- \| --- \| --- \|   TREATMENT ADMISSION IN SPECIALIZED MEDICAL CARE |
|  |

| 12. Did you have to take contact to health care after referral, before having the first appointment in specialized medical care? |
| --- |
| Yes |
| No |
| 12.1. If you answered yes, to what was the site you contacted? |
| The clinic I was referred to  Health services centre, emergency services |
| Hospital emergency department |
| Private health services |
| Some other, what? ___________________________  12.2. How many contacts did you have?  ____________________________________________ |
| 12.3. What was the reason for these contacts? Please write below informally |
| ______________________________________________________________ |
| ______________________________________________________________ |
| 12.4. Were there some additional actions based on these contacts? |
| My appointment in specialized medical care was made earlier  A new investigation was made |
| My referral was urged  A new referral to another speciality clinic |
| Some other, what?  ___________________________________________________ |

| 13. Time from referral to first appointment in specialized medical care |
| --- |
| _________ Date of first appointment in specialized medical care |
|  |
| In case you don’t remember the exact date, please make an estimate. (Estimate the time between you made an agreement or heard about the referral and had the first appointment in specialized medical care) |
| \| Immediately during the same day \| \| --- \| \| 1-2 days \| \| 3 -7 days \| \| 1 – 2 weeks \| \| 3 – 4 weeks \| \| 1 – 2 months  2 – 3 months  3 – 6 months \| \| Some other, what? ____________________ \| |

| 14. What was the speciality clinic where you had the first appointment? |
| --- |
| Internal medicine |
| Surgery |
| Otorhinolaryngology  Department of oncology or hematology |
| Emergency department, what speciality? ___________ |
| Neurosurgery  Some other, what? __________________________________ |
| 15. Date, when the biopsy was completed ____________________________ |

| 16. When did you hear the name of the disease (diagnosis) for the first time? (Please make an estimate if you don’t remember the exact date) |
| --- |
| ___________________________________________________ |

| 17. Who told you the diagnosis? (Occupation and speciality if you know it) |
| --- |
| ____________________________________________________ |
| How was the diagnosis told to you? |
| By letter |
| By phone |
| At the doctor’s appointment |
| Some other, how? _______________________________ |

| 18. When was your first appointment in the department of oncology or hematology? |
| --- |
| __________ Date |
| Please make an estimate if you don’t remember the exact date |
| _______________________________________________ |

| 19. When was your treatment initiated or it is planned to start? |
| --- |
| ____________ päivämäärä |

          

     

| 20. Is there something else in your mind concerning your admittance to treatment? |
| --- |
| __________________________________________________  __________________________________________________  __________________________________________________  __________________________________________________  __________________________________________________  __________________________________________________ |

                    

Thank you for your co-operation!
